# Optimal management for osteoporotic vertebral compression fractures: a network meta-analysis

**DOI:** 10.1186/s13018-025-06233-w

**Published:** 2025-08-30

**Authors:** Yan Li, Xianghong Wang, Jianfeng Sun, Maozhen Ma

**Affiliations:** 1 Department of Orthopedics, Xiyuan Hospital CACMS, Beijing, 100091 China; 2 Department of Surgery, West Campus Health Service Center CAU, Beijing, 100193 China; 3 Department of Orthopaedics, Lianshui People’s Hospital, Kangda College of Nanjing Medical University, Lianshui, 223400 Jiangsu China

**Keywords:** Osteoporotic vertebral compression fractures, Network meta-analysis, Efficacy, Safety, Non-surgical management, Surgical intervention

## Abstract

**Background:**

Optimal management of osteoporotic vertebral compression fractures (OVCFs) remains controversial. This network meta‑analysis (NMA) evaluated the relative efficacy and safety of third‑generation percutaneous vertebral augmentation (TVA), percutaneous kyphoplasty (PKP), percutaneous vertebroplasty (PVP), and non‑surgical management (NSM) in OVCFs.

**Methods:**

A systematic search of PubMed, Embase, the Cochrane Library, and Web of Science was conducted from inception to February 1, 2025, to identify clinical trials comparing ≥ 2 of these interventions. Primary outcomes included pain intensity (Visual Analog Scale [VAS]), functional disability (Oswestry Disability Index [ODI]), quality of life (EQ‑5D), and anterior vertebral body height (AVB). Adverse events, including adjacent vertebral fracture (AVF) and bone cement leakage (BCL), were also assessed.

**Results:**

Forty‑six studies (23 RCTs and 23 comparative cohort studies; *n* = 5,660) were included. Both TVA and PKP yielded greater VAS reductions than NSM at short‑term (≤ 6 months; MD − 1.28 and − 1.37; 95% CI − 1.62 to − 0.93 and − 1.82 to − 0.92) and long‑term (> 6 months; MD − 0.86 and − 0.69; 95% CI − 1.22 to − 0.50 and − 1.20 to − 0.19) follow‑up. TVA outperformed NSM in ODI improvement at short‑term (MD − 6.84; 95% CI − 9.84 to − 3.84) and long‑term (MD − 9.14; 95% CI − 14.64 to − 3.65); PKP surpassed NSM short‑term (MD − 5.59; 95% CI − 9.32 to − 1.86) but was inferior to TVA long‑term (MD 8.34; 95% CI 2.62 to 14.06). Surgical interventions uniformly outperformed NSM in quality‑of‑life gains. TVA and PKP achieved greater AVB restoration than NSM, whereas PVP carried a higher BCL risk compared to TVA and PKP. NSM was associated with the lowest probability of AVF (90.2%).

**Conclusions:**

Although PKP offers the greatest long‑term preservation of AVB, third‑generation TVA appears superior to PVP, PKP, and NSM in improving pain, functional disability, quality of life, and safety in OVCF patients. High‑quality randomized trials with extended follow‑up are required to confirm these findings.

**Supplementary Information:**

The online version contains supplementary material available at 10.1186/s13018-025-06233-w.

## Introduciton

Osteoporotic vertebral compression fractures (OVCFs) are common fractures in elderly individuals with osteoporosis, leading to significant morbidity characterized by pain, spinal deformity, impaired pulmonary function, and depression [1]. It is estimated that approximately 1.4 million new OVCFs occur worldwide annually, and up to 25% of people over 50 years of age will experience at least one vertebral fracture in their lifetime [2, 3]. With an aging global population, the incidence of OVCFs is expected to rise further, imposing a substantial economic burden [4]. For example, in 2005, the total annual cost of OVCFs in the United States exceeded US$1 billion; from 2006 to 2010, the average annual cost per patient in Germany was €6,490 (US$7,203); in Canada, the direct medical cost per patient reached CAD 25,965 (US$19,993) during 2011–2012; and in China, the annual direct medical cost per patient was RMB 21,474 (US$3,063) between 2010 and 2012 [5].

Alongside these interventional approaches, pharmacological management of osteoporosis has advanced markedly. Antiresorptive agents such as bisphosphonates have demonstrated sustained reductions in vertebral fracture risk [6], while denosumab—a RANKL inhibitor—provides continuous fracture protection and increases bone mineral density [6]. Anabolic therapies, including teriparatide and abaloparatide, not only stimulate new bone formation but also enhance vertebral microarchitecture, translating into lower fracture rates [7, 8]. More recently, romosozumab—an antisclerostin monoclonal antibody—has produced rapid gains in bone strength and superior vertebral fracture risk reduction compared to traditional agents over 12 months [10]. Collectively, these pharmacotherapies represent the cornerstone of fracture prevention and may favorably influence the biomechanical environment for subsequent surgical interventions [9, 10]. The primary treatment modalities for OVCFs include conservative management and surgical intervention. Conservative approaches—such as bed rest, bracing, immobilization, and pharmacotherapy—remain firstline, yet standardized care pathways are lacking, and randomized trials have questioned the superiority of vertebral augmentation over optimized nonsurgical management [11, 12]. In a landmark doubleblind RCT, vertebroplasty achieved more rapid and sustained pain relief and functional improvement compared to a regimen of three weeks’ bed rest plus hyperextension bracing, without increasing serious adverse events [13]. More recent trials indicate that both vertebroplasty and balloon kyphoplasty provide significant and comparable pain relief, whereas kyphoplasty more effectively restores vertebral height and reduces cement leakage rates [12–14]. Surgery is indicated for patients who fail to respond to nonsurgical management or who have persistent pain after three weeks [15]. Surgical options include percutaneous vertebroplasty (PVP), percutaneous kyphoplasty (PKP), and thirdgeneration vertebral augmentation (TVA) systems—such as SpineJack, radiofrequency kyphoplasty, Kiva, SKY, and vertebral body stenting [16–20]. However, some studies report no superiority of augmentation over conservative care and note an increased risk of complications—particularly adjacent vertebral fractures and bone cement leakage [3, 21, 22]—with augmentation procedures [23–26]. TVA devices employ an expandable scaffold to more effectively restore vertebral height before cement injection, potentially offering greater symptomatic relief than PVP or PKP. Nevertheless, trials comparing TVA with PVP and PKP have yielded inconsistent results regarding symptom recovery, height restoration, and adverse event rates [20, 27, 28]. Thus, the optimal interventional strategy for OVCFs remains controversial.

Because direct headtohead comparisons are limited, traditional pairwise metaanalyses cannot fully resolve these questions. Network metaanalysis (NMA) extends conventional methods by combining direct and indirect evidence across multiple interventions, thereby enhancing inference on relative treatment efficacy [29–31]. Here, we present the first comprehensive NMA evaluating the safety and efficacy of NSM, PVP, PKP, and TVA systems to identify optimal treatment modalities for OVCFs. The objective of this study is to provide evidencebased recommendations for clinical practice in managing OVCFs and to alleviate the substantial societal burden associated with these fractures.

## Methods

### Search strategy

This network metaanalysis was conducted in accordance with PRISMA (Preferred Reporting Items for Systematic Reviews and MetaAnalyses) and AMSTAR (Assessing the Methodological Quality of Systematic Reviews) guidelines [32–34], and the protocol was registered in PROSPERO. We systematically searched PubMed, Embase, Web of Science, the Cochrane Library, and major scientific databases from inception through February 1, 2024, for randomized controlled trials (RCTs), comparative studies, and relevant reviews reporting at least two different interventions. Search terms included “vertebral fractures,” “vertebral compression fractures,” and “osteoporotic vertebral compression fractures.” No restrictions were applied to language or publication status. Reference lists of all eligible studies were handsearched to identify additional reports.

### Inclusion and exclusion criteria

Studies were included if they met the following criteria: (1) RCTs or comparative studies involving ≥ 2 interventions; (2) adult patients diagnosed with OVCFs; and (3) reporting ≥ 1 primary outcome measure. Exclusion criteria were: (1) case reports, conference proceedings, and reviews; (2) patients with comorbid injuries, tumors, or other conditions significantly affecting quality of life; and (3) patients with prior vertebral surgery or revision procedures.

### Follow-up definitions

Follow‑up intervals were predefined as short‑term (≤ 6 months) and long‑term (> 6 months). Although a 12‑month threshold was considered, only five studies reported outcomes at or beyond one year, preventing robust comparisons at that cut-off. Therefore, we retained the 6‑month boundary to ensure consistency and statistical power.

### Outcome measures and data extraction

Primary outcomes included: (1) pain intensity, assessed by the visual analogue scale (VAS); (2) functional disability, assessed by the Oswestry Disability Index (ODI); (3) radiological outcome, defined as anterior vertebral body height (AVB); and (4) complications, including adjacent vertebral fracture (AVF) and bone cement leakage (BCL). The secondary outcome was quality of life, measured by the EuroQol‑5 Dimensions questionnaire (EQ‑5D). Data extraction was performed independently by two reviewers (Y.L. and X.H.W.) using a standardized form to capture publication details, study design, intervention types, follow‑up duration, and outcome data. A second search was conducted post‑extraction to ensure completeness.

### Study quality assessment

Two reviewers (J.F.S. and M.Z.M.) independently assessed risk of bias. The Cochrane Risk of Bias tool was applied to RCTs [32], and the Newcastle–Ottawa Scale (NOS) was used for cohort studies [35]. Discrepancies were resolved by consensus.

### Statistical analysis

Continuous outcomes were summarized as mean differences (MDs) with 95% confidence intervals (CIs), and dichotomous outcomes were expressed as odds ratios (ORs) with 95% CIs. A p‑value < 0.05 was considered statistically significant. Heterogeneity was evaluated using Cochran’s Q and I^2^ statistics (*P* < 0.1 and I^2^ > 50% indicating substantial heterogeneity) [36]. A random‑effects model was used when heterogeneity was significant; otherwise, a fixed‑effects model was applied.

Network geometry plots were generated to visualize direct comparisons. Under a frequentist consistency framework, direct and indirect evidence were combined to estimate relative intervention effects; if no direct comparison existed, estimates were derived from indirect evidence alone [37, 38]. Results were presented in league tables (ladder diagrams), and intervention rankings were determined by the surface under the cumulative ranking curve (SUCRA). Inconsistency between direct and indirect evidence was assessed by comparing deviance information criteria and by node‑splitting analysis [37, 39]. Publication bias was evaluated using funnel plots and Egger’s test. All analyses were performed using STATA version 17.

## Results

### Systematic review and qualitative assessment

**Fig. 1:**
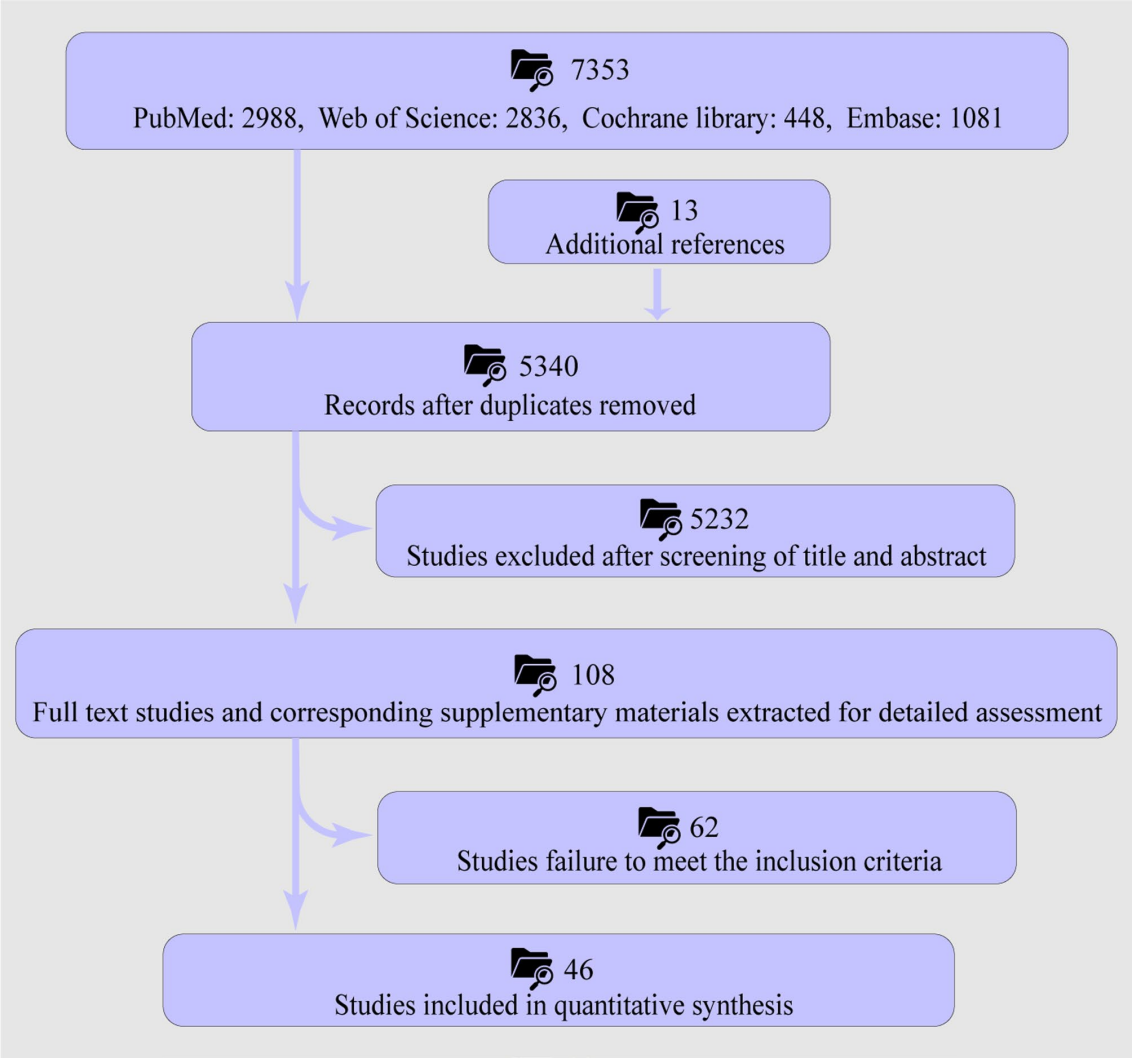
Flow diagram of study selection process and overall design

Figure 1 illustrates the selection process and primary reasons for exclusion. Fortysix trials [2, 3, 16–20, 27, 28, 40–76] (23 RCTs and 23 cohort studies; *n* = 5,660) from 20 countries (China 11; Germany 7; Australia 4; USA 3; Spain 3; Italy 2; Austria 2; Netherlands 2; Canada, Slovenia, India, Belgium, Denmark, Greece, Iran, Israel, Japan, Singapore, South Korea, and Switzerland each 1) comparing at least two interventions (NSM, PKP, PVP, and TVA) were included (Supplementary Table 1). All studies were published as full manuscripts. Risk-of‐bias assessment (Cochrane ROB for RCTs; NOS for cohort studies) indicated generally low risk in the majority of trials (Supplementary Figs. 1–2; Supplementary Table 2). Followup intervals were defined a priori as shortterm (≤ 6 months) or longterm (> 6 months).

### Pain relief (VAS)

**Fig. 2:**
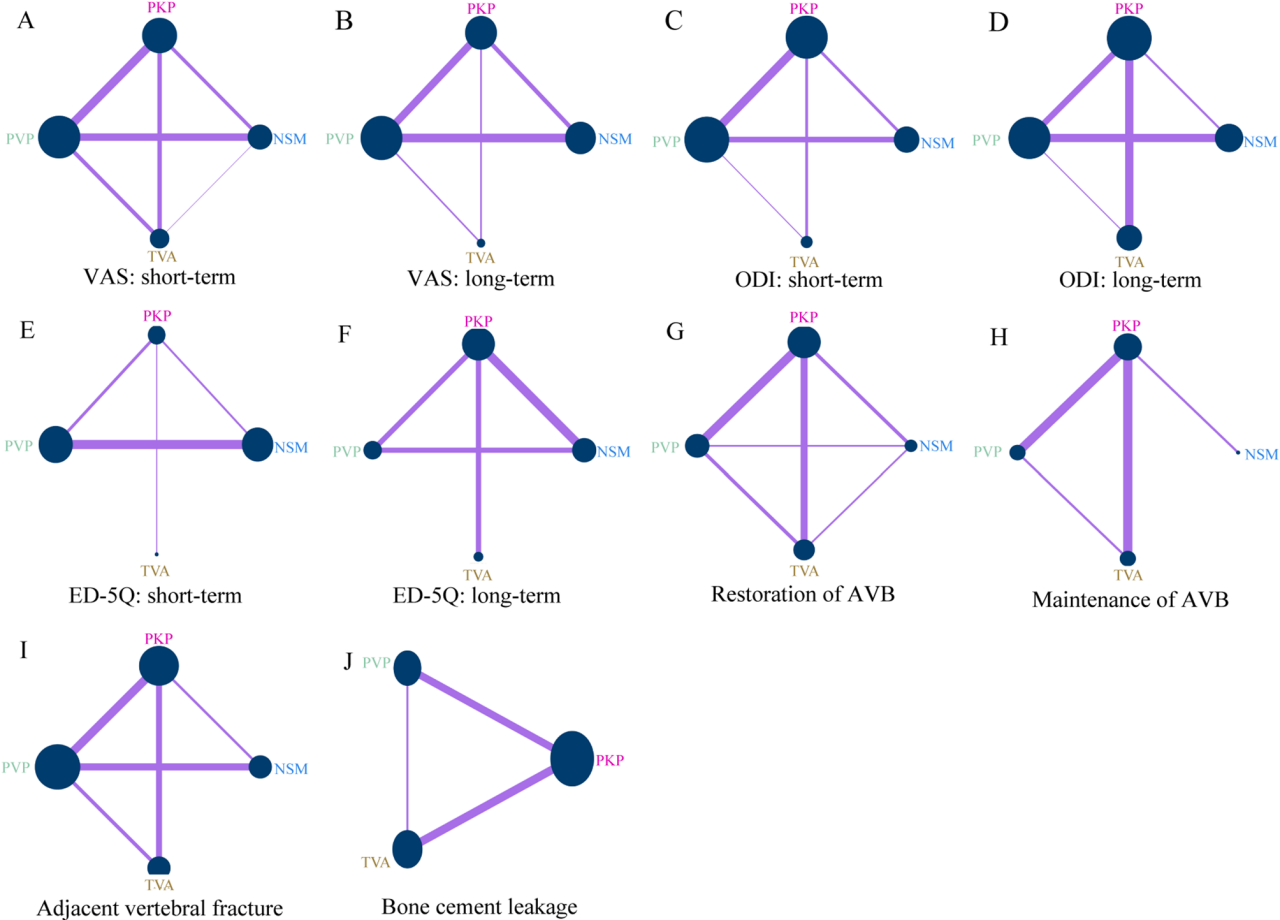
Network geometry plots for the comparison-based network meta-analysis. Each node represents an intervention; its area is proportional to the total sample size, and each connecting line’s thickness corresponds to the number of head-to-head trials. (NSM: non-surgical management; PKP: percutaneous kyphoplasty; PVP: percutaneous vertebroplasty; TVA: third-generation vertebral augmentation system.)

**Fig. 3:**
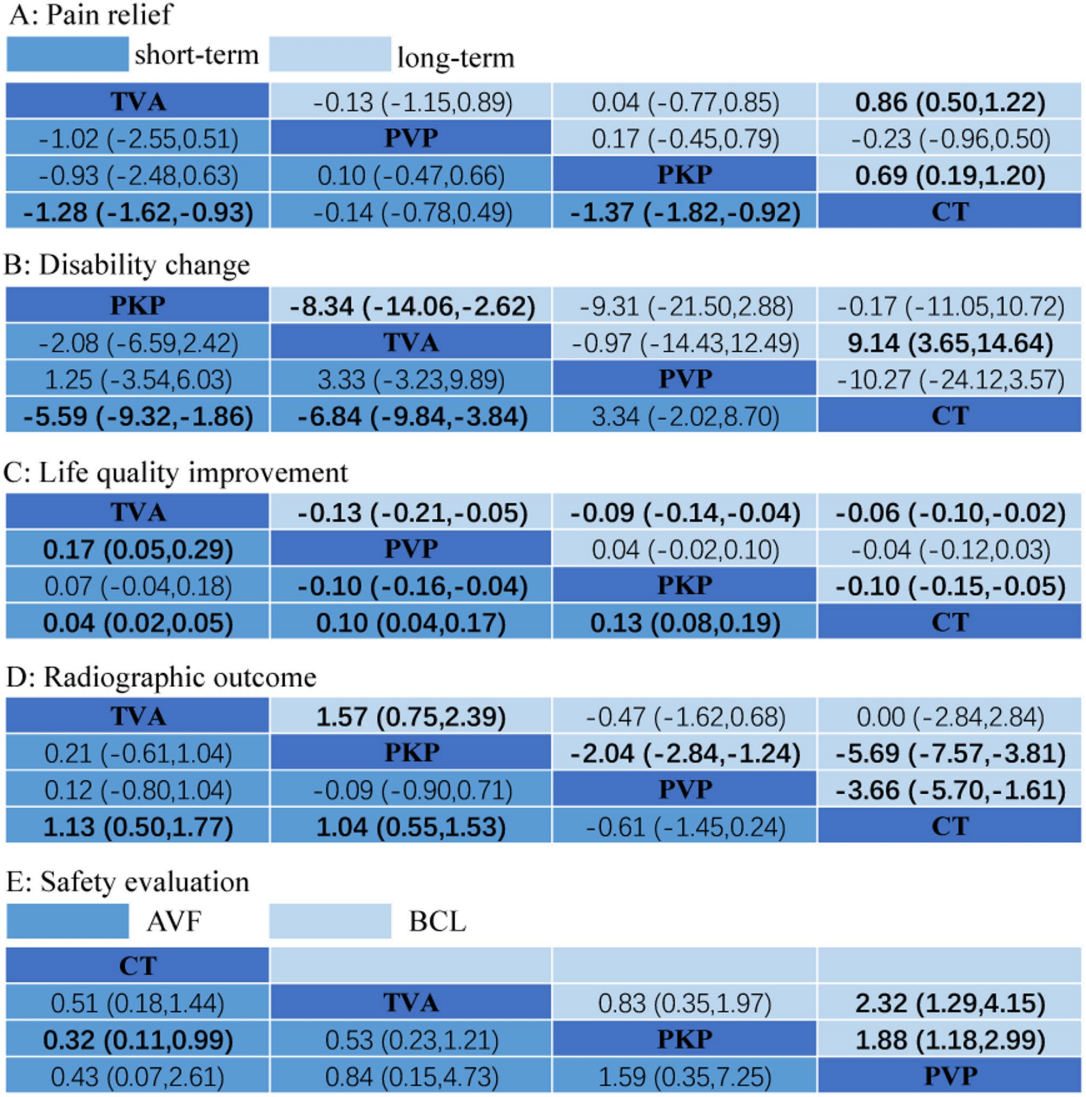
League tables of network meta-analysis outcomes. Each cell shows the pooled mean difference with 95% confidence interval; statistically significant comparisons are highlighted in bold. (NSM: non-surgical management; PKP: percutaneous kyphoplasty; PVP: percutaneous vertebroplasty; TVA: third-generation vertebral augmentation system.)

**Fig. 4:**
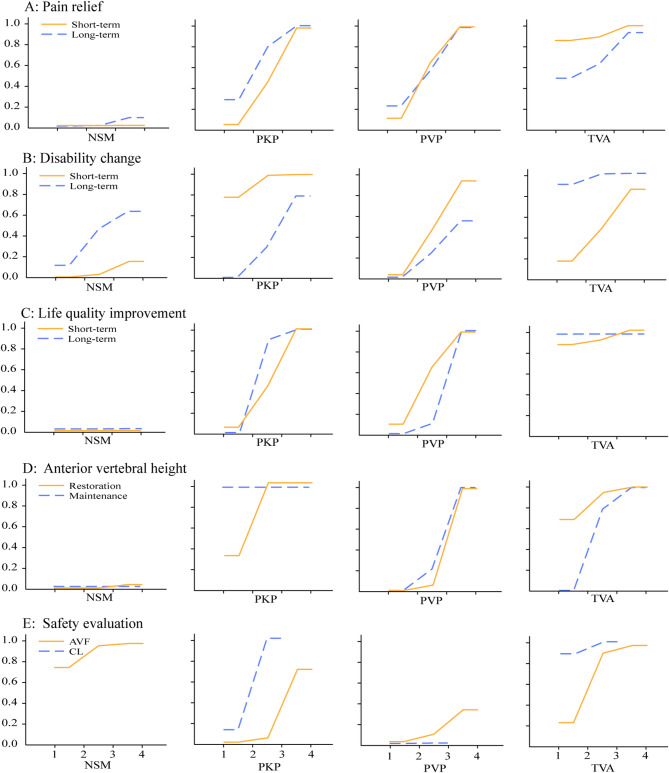
Surface under the cumulative ranking curve (SUCRA) plots. Higher SUCRA values indicate greater likelihood of an intervention being the most effective. (NSM: non-surgical management; PKP: percutaneous kyphoplasty; PVP: percutaneous vertebroplasty; TVA: third-generation vertebral augmentation system.)

Thirty studies [2, 16, 18, 40–44, 46, 49–51, 54–61, 64–72, 76] (*n* = 4,305) reported VAS outcomes for NSM, TVA, PKP, and PVP. Network geometry is shown in Fig. 2A–B. Under the consistency model (Fig. 3A), both TVA and PKP achieved significantly greater pain reduction versus NSM in the shortterm (MD − 1.28 and − 1.37; 95% CI − 1.62 to − 0.93 and − 1.82 to − 0.92) and longterm (MD − 0.86 and − 0.69; 95% CI − 1.22 to − 0.50 and − 1.20 to − 0.19). Inconsistency checks, including nodesplitting, revealed no significant discrepancies (all *p* > 0.05; Supplementary Table 3). SUCRA rankings (Fig. 4A) indicated that TVA had the highest probability of being most effective for shortterm pain relief (91.3%), followed by PVP (58.3%), PKP (50.4%), and NSM (0%). For longterm relief, the ranking was PKP (69.3%), TVA (68.8%), PVP (58.8%), and NSM (3.1%).

### Functional disability (ODI)

Fifteen studies [17, 28, 40, 43, 47, 50, 51, 54, 56, 57, 59, 67, 71, 72, 76] (*n* = 2,372) reported ODI changes. Network plots appear in Fig. 2C–D. TVA demonstrated significantly greater ODI improvement than NSM in both shortterm and longterm followup (MD − 6.84 and − 9.14; 95% CI − 9.84 to − 3.84 and − 14.64 to − 3.65). PKP outperformed NSM shortterm (MD − 5.59; 95% CI − 9.32 to − 1.86) but was ultimately surpassed by TVA longterm (MD 8.34; 95% CI 2.62 to 14.06). Consistency and inconsistency models were in agreement (all *p* > 0.05; Supplementary Table 4). SUCRA rankings (Fig. 4B) identified PKP as most effective for shortterm disability improvement (92.3%), followed by TVA (52.5%), PVP (49.1%), and NSM (6.1%); for longterm improvement, TVA ranked highest (96.2%), followed by NSM (38.9%), PKP (36.8%), and PVP (28.1%).

### Quality of life (EQ‑5D)

Ten studies [3, 27, 40, 47, 53, 60, 61, 65, 66, 73] (*n* = 1,170) assessed EQ-5D. Network geometry is shown in Fig. 2E–F. Surgical interventions generally improved shortterm EQ-5D versus NSM (TVA MD 0.04; PVP 0.10; PKP 0.13; 95% CI 0.02–0.05, 0.04–0.17, 0.08–0.19), and TVA and PKP outperformed PVP (MD 0.17 and 0.10; 95% CI 0.05–0.29, 0.04–0.16). In the long term, TVA led to the greatest improvement (MD 0.13, 95% CI 0.05–0.21), followed by PKP (0.09; 0.04–0.14) and PVP (0.06; 0.02–0.10). PKP also exceeded NSM longterm (MD 0.10; 0.05–0.15). Consistency and inconsistency models agreed for longterm outcomes (all *p* > 0.05; Supplementary Table 5); some shortterm comparisons showed inconsistency. SUCRA probabilities (Fig. 4C) ranked shortterm efficacy as TVA (92.1%), PVP (58.6%), PKP (49.2%), NSM (0%), and longterm as TVA (99.9%), PKP (63.3%), PVP (36.6%), NSM (0.1%).

### Anterior vertebral body height (AVB)

Fourteen studies [18, 28, 40, 41, 44, 49–52, 54, 64, 69, 71, 75] (*n* = 1,398) evaluated AVB restoration and maintenance. Network plots appear in Fig. 2G–H. TVA and PKP both restored AVB significantly more than NSM (MD 1.13 and 1.04; 95% CI 0.50–1.77, 0.55–1.53). Longterm AVB maintenance was greatest with PKP (MD 1.57 vs. TVA; 2.04 vs. PVP; 5.69 vs. NSM; all *p* < 0.05), and PVP also outperformed NSM (MD 3.66; 1.61–5.70). Consistency and inconsistency models agreed for maintenance (all *p* > 0.05; Supplementary Table 6), though some restoration comparisons were inconsistent. SUCRA (Fig. 4D) ranked restoration as TVA (88.0%), PKP (76.9%), PVP (34.0%), NSM (1.1%), and maintenance as PKP (100.0%), TVA (59.7%), PVP (40.3%), NSM (0.0%).

### Adverse events

#### Adjacent vertebral fracture

Twentythree studies [3, 17, 18, 27, 28, 41, 43–48, 53–55, 58, 59, 63–65, 68, 72, 73] (*n* = 2,366) compared AVF rates. Network geometry is shown in Fig. 2I. NSM exhibited a significantly lower AVF rate than PKP (OR 0.32; 95% CI 0.11–0.99), while no other pairwise differences reached significance (Fig. 3E). Consistency and inconsistency models were identical, and nodesplitting revealed no significant inconsistency (all *p* > 0.05; Supplementary Table 7). SUCRA indicated NSM as least likely to cause AVF (90.2%; Fig. 4E).

#### Bone cement leakage

Twenty studies [17, 19, 20, 27, 28, 43, 45–48, 50–52, 63, 64, 71–74, 76] (*n* = 2,785) reported BCL rates. Network plots appear in Fig. 2J. Among surgical procedures, PVP had higher BCL risk than TVA (OR 2.32; 95% CI 1.29–4.15) and PKP (OR 1.18; 95% CI 1.18–2.99) (Fig. 3E). Consistency and inconsistency models agreed, with no significant nodesplitting inconsistencies (all *p* > 0.05; Supplementary Table 7). SUCRA rankings (Fig. 4E) identified TVA (94.6%) as least likely to result in BCL, followed by PKP (55.1%) and PVP (0.3%).

No significant publication bias was detected by Begg’s or Egger’s tests (all *p* > 0.05).

## Discussion

This study represents the first comprehensive NMA to identify the optimal treatment for OVCFs. We included twenty‑three randomized controlled trials and twenty‑three comparative cohort studies—each evaluating at least two interventions (NSM, PVP, PKP, and TVA). In our systematic review and NMA, we prioritized both short‑term (≤ 6 months) and long‑term (> 6 months) outcomes—pain, functional disability, quality of life, and radiographic parameters—given their established importance in assessing the efficacy and safety of these treatments. Crucially, our comparative evaluation of vertebral augmentation should be interpreted in the context of advances in osteoporosis pharmacotherapy: bisphosphonates, denosumab, teriparatide, and romosozumab reduce incident vertebral fractures by 50–70% and enhance the bone microenvironment that underlies surgical success. Future trials ought to investigate combined protocols of optimized medical regimens with device‑based augmentation to maximize spinal health in osteoporotic patients.

The results of our NMA indicate that third‑generation TVA delivers superior short‑term outcomes—significantly greater reductions in VAS pain scores, improvements in ODI‑measured functional disability, and enhancements in EQ‑5D‑assessed quality of life—compared with PKP, PVP, and NSM. Over the long term, TVA maintained its advantage in reducing ODI scores, whereas PKP provided the greatest benefit in preserving anterior vertebral body height (AVB). Notably, no severe adverse events were reported among surgically treated patients. Although PKP excelled in long‑term AVB maintenance, our findings suggest that TVA offers the most balanced profile of analgesia, functional restoration, quality‑of‑life improvement, and safety. Well‑designed randomized trials with extended follow‑up are warranted to confirm these conclusions. Recent studies have further refined our understanding of post‑augmentation outcomes and patient‑level risk stratification: Wang et al. described an accelerated cascade of acute multiple OVCFs—predominantly at L1—with adjacent‑level progression of nearly one new fracture per two vertebrae per year [77]. Wu et al. applied machine‑learning models to predict residual back pain (VAS ≥ 4) one month post‑vertebroplasty, identifying cement volume and intravertebral vacuum clefts as key predictors [78]. Tang et al. reported that low baseline bone mineral density, excessive kyphotic angle correction, and adjacent cement leakage significantly increase secondary fracture risk after augmentation [79]. Finally, Wang et al. presented preliminary data from sham‑controlled vertebroplasty trials, highlighting substantial placebo effects on analgesia and underscoring the necessity for rigorous blinding in future studies [80].

Nonsurgical management remains a viable option for patients with mild symptoms, minimal radiographic compression, intact neurological function, or contraindications to surgery. Although conservative therapy can provide symptom relief in some cases, most patients ultimately derive greater benefit from surgical intervention [2, 57, 81]. Our NMA indicates that thirdgeneration TVA may offer superior efficacy in OVCF treatment compared to PVP, PKP, and NSM, without an increase in severe adverse events. Over the past decade, the adoption of thirdgeneration TVA has grown substantially. While PVP— the inaugural augmentation technique—provides rapid analgesia and spinal stabilization via polymethylmethacrylate injection into the intertrabecular space, it is limited by suboptimal height restoration and bone cement leakage rates up to 54.7%, consistent with our findings [82]. PKP improves height restoration but is associated with a higher refracture risk in cemented vertebrae, particularly in the presence of intravertebral clefts [82]. These limitations motivated the development of thirdgeneration TVA, which employs an expandable scaffold permanently implanted in the vertebral body to achieve mechanical reduction before cement delivery. This approach facilitates enhanced restoration of vertebral height and, consequently, superior clinical symptom improvement. Notably, our results corroborate these mechanistic advantages observed in prior studies.

Regarding adverse events, AVFs and BCL are the most frequent and serious complications following surgical treatment of OVCFs, substantially impacting patient prognosis. Surgical reinforcement of the treated vertebra increases its stiffness, thereby transferring biomechanical loads to adjacent levels and raising AVF risk [83, 84]. Consistent with this mechanism, our NMA found that NSM was associated with a significantly lower AVF rate compared with PKP, and SUCRA rankings indicated that all augmentation procedures carry a higher AVF risk than NSM. Although SUCRA probabilities are indicative rather than definitive, they offer valuable comparative insights. Conversely, BCL can be lifethreatening if cement extravasates into the spinal canal or venous circulation; intravascular cement is particularly prone to cause pulmonary embolism and sudden death [85–87]. Our analysis demonstrated that PVP had the highest BCL incidence among surgical techniques—likely due to its direct cement injection without cavity creation—whereas PKP’s balloon tamp mitigates leakage. Importantly, thirdgeneration TVA did not increase BCL risk relative to PVP, PKP, or NSM, further underscoring its safety advantage in OVCF management.

However, several limitations of this NMA warrant consideration. First, although we prioritized randomized controlled trials, a proportion of the included studies were non-randomized comparative designs, which may introduce confounding and bias into the network estimates. Second, our use of a 6-month threshold to define long-term outcomes may not fully capture intermediate effects, and the inclusion of three studies with only 3-month follow-up could contribute to early-time heterogeneity. Third, the “third-generation TVA” category encompasses multiple scaffold systems (e.g., SpineJack, Kiva, VBS, RFK) with different deployment mechanics; pooling these devices may obscure scaffold-specific effects on anterior vertebral body height maintenance. Fourth, several trials had relatively small sample sizes, potentially reducing statistical power and increasing uncertainty around effect estimates. Fifth, variability in baseline patient characteristics (e.g., age, fracture severity) and differences in follow-up assessment protocols (imaging modality and measurement timing) may have contributed to heterogeneity across comparisons. Finally, despite rigorous inclusion criteria, slight variations in surgical technique, cement viscosity, and operator expertise among the studies may limit the external validity of our findings.

## Conclusions

This comprehensive network meta‑analysis of OVCF treatments demonstrates that third‑generation TVA offers the most balanced benefit profile—providing superior short‑ and long‑term improvements in pain, functional disability, and quality of life—despite PKP’s advantage in long‑term anterior vertebral body height maintenance. Given the heterogeneity and methodological limitations of the available studies, as well as the relative paucity of high‑quality randomized trials, further well‑designed RCTs are essential to confirm and refine these findings.

## Supplementary Information

Below is the link to the electronic supplementary material.

Supplementary Material 1

## Data Availability

All data generated or analysed during this study are included in this published article and its supplementary information files.

## Abbreviations

OVCFs: osteoporotic vertebral compression fractures
NMA: network meta-analysis
TVA: third-generation vertebral augmentation system
PKP: percutaneous kyphoplasty
PVP: percutaneous vertebroplasty
NSM: non-surgical treatment
AVB: anterior vertebral height
RCTs: randomized controlled trials
VAS: visual analogue scale
ODI: Oswestry Disability Index
AVF: adjacent vertebral fracture
BCL: bone cement leakage
ED-5Q: EuroQol-5-Domain questionnaire
MD: mean difference
CI: confidence interval
OR: odds ratio
SUCRA: surface under the cumulative ranking curve
