# Supplementary material for: Optimal management for osteoporotic vertebral compression fractures: a network meta-analysis
Source: J Orthop Surg Res. 2025 Aug 30;20:810. doi: 10.1186/s13018-025-06233-w (PMC12398152; doi:10.1186/s13018-025-06233-w)
Supplement: Supplementary file 1 — Supplementary Material 1 [file 13018_2025_6233_MOESM1_ESM.docx]

**Table. S1 Basic data.**

| **No.** | **Study** | **Country** | **Age(Male/Female)** | **Degree of Fracture** | **Treatment/Comparison** | **Follow-up time** | **Outcomes** |
| --- | --- | --- | --- | --- | --- | --- | --- |
| **PVP vs PKP** | | | | | | | |
| 1 | M.Dohm et al, 2014 | USA, RCT | 76.6 (M/F=50/141) | 150 one level, 41 multiple levels | PVP(n=91)/PKP(n=100) | 24 months | VAS, ODI, SF-36, ED-5Q, QUALEFFO-41, Complications, Operation time, Vertebral body height, Kyphotic angle, Cobb angle |
| 2 | Jung-Tung Liu et al,2014 | China, RCT | PVP/PKP= 72.3/74.3 (M/F=23/77) | one level | PVP(n=50)/PKP(n=50) | 60 months | VAS, Complications, Operation time, Vertebral body height, Kyphotic angle |
| 3 | Avery J Evans et al, 2015 | USA, RCT | 75.6 (M/F=33/82) | 41 one level, 74 multiple levels | PVP(n=56)/PKP(n=59) | 12 months | VAS, RMDQ |
| 4 | C.Griffoni et al,2020 | Italy, RCT | PVP/PKP= 72/75 (M/F=20/93) | 75 one level, 39 multiple levels | PVP(n=64)/PKP(n=49) | 12 months | VAS, ODI, Complications, Kyphotic angle |
| 5 | JT Liu et al, 2010 | China, RCT | PVP/PKP=  74.3/72.3 (M/F=23/77) | one level | PVP(n=50)/PKP(n=50) | 6 months | VAS, Complications, Operation time, Vertebral body height, Kyphotic angle |
| 6 | Alessio Lovi et al, 2009 | Italy, prospectively | 67.6 (M/F=56/98) | 50 one level, 104 multiple levels | PVP(n=118)/PKP(n=36) | 33 months | Complications, Operation time |
| 7 | Markus Dietmar Schofer et al, 2009 | Germany, prospectively | PVP/PKP=  73.8/72.5 (M/F=14/46) | one level | PVP(n=30)/PKP(n=30) | PVP/PKP=  13.7/13.5 months | VAS, Complications, Kyphotic angle |
| 8 | Krishna Kumar et al, 2010 | Canada, prospectively | PVP/PKP=  78/73  (M/F=16/36) | 23 one level, 29 multiple levels | PVP(n=28)/PKP(n=24) | PVP/PKP= 42.2/42.3 months | VAS, ODI, ED-5Q, Complications |
| 9 | I.Movrin et al, 2010 | Slovenia, prospectively | PVP/PKP=  72.9/67.8 (M/F=15/58) | 64 one level, 9 multiple levels | PVP(n=27)/PKP(n=46) | 12 months | Complications, Compression rate, Kyphotic angle |
| 10 | Renbin Dong et al. 2012 | China, prospectively | 70.1 (M/F=34/52) | 78 one level, 8 multiple levels | PVP(n=35)/PKP(n=51) | 21.3 months | VAS, Vertebral body height, Kyphotic angle |
| 11 | K-Z Hu et al, 2018 | China, prospectively | PVP/PKP=  71.4/70.6 (M/F=62/99) | one level | PVP(n=70)/PKP(n=91) | 25 months | VAS, ODI, Complications, Vertebral body height, Cobb angle |
| 12 | Yi Zhou et al, 2022 | China, prospectively | PVP/PKP=  63.5/64.7 (M/F=66/48) | 138 vertebral bodies | PVP(n=56)/PKP(n=58) | 6 months | VAS, ODI, Complications, Operation time, Vertebral body height |
| 13 | Vasu Jindal et al,2022 | India, prospectively | 56.375 (M/F=15/25) | one level | PVP(n=20)/PKP(n=20) | 6 months | Complications, Operation time, Vertebral body height |
| **PVP vs NSM** | | | | | | | |
| 14 | Caroline A H Klazen et al,2010 | Netherlands, RCT | PVP/CT=  75.2/75.4 (M/F=62/140) | 265 vertebral bodies | PVP(n=101)/CT(n=101) | 11.4 months | VAS, Complications |
| 15 | Rikke Rousing et al, 2010 | Denmark, RCT | 80 (M/F=9/40) | 37 one level, 12 multiple levels | PVP(n=25)/CT(n=24) | 12 months | VAS, SF-36, ED-5Q, Complications |
| 16 | Majid Reza Farrokhi et al,2011 | Iran, RCT | PVP/CT=  72/74 (M/F=22/60) | 190 vertebral bodies | PVP(n=40)/CT(n=42) | 15 months | VAS, ODI, Complications, Vertebral body height, Kyphotic angle |
| 17 | Jordi Blasco et al, 2012 | Spain, RCT | PVP/CT=  71.33/75.27 (M/F=18/97) | 92 multiple levels | PVP(n=64)/CT(n=61) | 12 months | VAS, QUALEFFO-41, Complications |
| 18 | Biao Wang et al, 2016 | China, RCT | PVP/CT=  63.68/62.59 (M/F=41/165) | one level | PVP(n=100)/CT(n=106) | 12 months | VAS, ODI, RMDQ, SF-36, Complications, Operation time |
| 19 | Er-Zhu Yang et al, 2016 | China, RCT | PVP/CT=  77.1/76.2 (M/F=38/69) | 91 one level, 16 multiple levels | PVP(n=56)/CT(n=51) | 12 months | VAS, ODI, QUALEFFO-41, Complications |
| 20 | Terrence H Diamond et al, 2006 | Australia, prospectively | PVP/CT=  76.8/76.1 (M/F=39/87) | 46 one level, 80 multiple levels | PVP(n=88)/CT(n=38) | 24 months | VAS, Complications |
| 21 | Hao-Kuang Wang et al, 2010 | China, prospectively | PVP/CT=  72.9/72.7  (M/F=8/47) | 68 vertebral bodies | PVP(n=32)/CT(n=23) | 12 months | VAS, ODI, Complications |
| 22 | Rachelle Buchbinder et al,2009 | Australia, RCT | PVP/Sham=  74.2/78.9 (M/F=16/62) | 39 one level, 39 multiple levels | PVP(n=38)/sham(n=40) | 6 months | VAS, RDQ, AQoL, ED-5Q, QUALEFFO-41, Complications |
| 23 | Bryan A.Comstock et al,2013 | USA, RCT | 73.8 (M/F=42/99) | 99 one level, 42 multiple levels | PVP(n=68)/sham(n=63) | 12 months | VAS, RDQ, SF-36, ED-5Q |
| 24 | William Clark et al, 2016 | Australia, RCT | PVP/sham=  80/81  (M/F=32/88) | 104 one level, 16 multiple levels | PVP(n=61)/sham(n=59) | 6 months | VAS, RDQ, ED-5Q, QUALEFFO-41 |
| 25 | Cristina E Firanescu et al, 2018 | Netherlands, RCT | PVP/sham=  74.7/76.9 (M/F=18/133) | 137 one level, 39 multiple levels | PVP(n=90)/sham(n=86) | 12 months | VAS |
| **PVP vs TVA** | | | | | | | |
| 26 | Yoram Folman et al,2011 | Israel, prospectively | PVP/SKY=  75.6/70.7 (M/F=14/31) | one level | PVP(n=14)/SKY(n=31) | 12 months | VAS, Kyphotic angle |
| 27 | Martin Thaler et al, 2013 | Austria, prospectively | PVP/VBS=  67.9/66.9  (M/F=7/49) | 34 one level, 22 multiple levels | PVP(n=29)/VBS(n=27) | 3.1 months | Complications |
| 28 | Jiann-Her Lin et al, 2016 | China, retrospectively | PVP/SJ=  75.73/72.62 (M/F=10/65) | one level | PVP(n=39)/SJ(n=36) | 12 months | VAS, Complications, Vertebral body height, Kyphotic angle |
| 29 | Chi-Chen Huang et al, 2020 | China, retrospectively | PVP/SJ=  73.59/71.62 (M/F=21/53) | one level | PVP(n=32)/SJ(n=42) | 3 months | VAS, Complications, Compression rate, Vertebral body height, Kyphotic angle, Cobb angle |
| **PKP vs NSM** | | | | | | | |
| 30 | Steven Boonen et al, 2011 | Belgium, RCT | PKP/CT=  72.2/74.1 (M/F=68/232) | 215 one level, 85 multiple levels | PKP(n=149)/CT(n=151) | 24 months | VAS, RMDQ, SF-36, ED-5Q, Complications |
| 31 | Feline Kroon et al, 2014 | Australia, RCT | PKP/sham=  76.7/77.7 (M/F=10/47) | 50 one level, 28 multiple levels | PKP(n=38)/sham(n=40) | 24 months | VAS, RDQ, AQoL, ED-5Q, QUALEFFO-41 |
| 32 | Hwan Mo Lee et al, 2012 | South Korea, prospectively | PKP/CT=  76.8/66.2 (M/F=90/141) | 182 one level, 49 multiple levels | PKP(n=82)/CT(n=149) | 12 months | VAS, ODI |
| 33 | Masatoshi Hoshino et al, 2018 | Japan, prospectively | PKP/CT=  78.5/77.4 (M/F=26/154) | one level | PKP(n=90)/CT(n=90) | 6 months | VAS, SF-36, Complications, Cobb angle |
| 34 | Dejun Yu et al, 2022 | China, prospectively | PKP/CT=  67.07/67.03 (M/F=27/33) | one level | PKP(n=30)/CT(n=30) | 6 months | VAS, SF-36, Vertebral body height |
| **PKP vs TVA** | | | | | | | |
| 35 | H-J Riesner et al, 2016 | Germany, RCT | 78.5 (M/F=24/76) | 162 vertebral bodies | PKP(n=48)/RFK(n=53) | NA | Complications |
| 36 | R Pflugmacher et al,2012 | Germany, prospectively | PKP/RFK=  65.2/70.1 (M/F=89/139) | 127 one level, 101 multiple levels | PKP(n=114)/RFK(n=114) | 6 months | VAS |
| 37 | Rahel Bornemann et al, 2017 | Germany, retrospectively | PKP/RFK=  67.9/69.6 (M/F=76/116) | 108 one level, 84 multiple levels | PKP(n=96)/RFK(n=96) | 24 months | VAS, ODI, Complications, Vertebral body height, Kyphotic angle |
| 38 | Panagiotis Korovessis et al,2013 | Greece, RCT | PKP/KIVA=  72.3/69.6 (M/F=49/119) | 120 one level, 48 multiple levels | PKP(n=86)/KIVA(n=82) | 14 months | VAS, ODI, SF-36, Complications, Operation time, Kyphotic angle |
| 39 | Sean M. Tutton et al, 2015 | Germany, RCT | PKP/KIVA=  75.09/76.03 (M/F=74/211) | 215 one level, 70 multiple levels | PKP(n=144)/KIVA(n=141) | 12 months | VAS, ODI, Complications |
| 40 | Lucia A. Otten et al, 2013 | Germany, prospectively | PKP/KIVA=  66.4/73.6 (M/F=17/35) | 36 one level, 16 multiple levels | PKP(n=26)/KIVA(n=26) | 6 months | VAS, ODI, Complications, Operation time, Vertebral body height |
| 41 | D.C.Noriega et al, 2019 | Spain, RCT | PKP/SJ=  68.3/67.9 (M/F=6/24) | 27 one level, 3 multiple levels | PKP(n=15)/SJ(n=15) | 36 months | VAS, ODI, ED-5Q, Complications, Operation time |
| 42 | David Noriega et al,2019 | Spain, RCT | PKP/SJ=  72.2/74.4 (M/F=30/111) | 127 one level, 14 multiple levels | PKP(n=73)/SJ(n=68) | 12 months | VAS, ODI, ED-5Q, Complications, Operation time |
| 43 | Clement M.L.Werner et al, 2013 | Switzerland, RCT | 70 (M/F=25/40) | 100 vertebral bodies | PKP(n=25)/VBS(n=40) | NA | Complications |
| 44 | Sebastian Schutzenberger et al,2018 | Austria, retrospectively | PKP/VBS=  69.2/68.5 (M/F=14/35) | one level | PKP(n=13)/VBS(n=36) | 24 months | VAS, ODI, Complications, Operation time, Kyphotic angle, Cobb angle |
| **TVA vs NSM** | | | | | | | |
| 45 | Rahel Bornemannet al, 2012 | Germany, prospectively | 65 (M/F=36/35) | 44 one level, 71 multiple levels | RFK(n=33)/CT(n=38) | 3 months | VAS, ODI, Complications, Vertebral body height, Kyphotic angle |
| **PVP vs PKP vs TVA vs NSM** | | | | | | | |
| 46 | Gerard Wen Wei Ee et al, 2015 | Singapore, retrospectively | PVP/PKP/SKY/CT= 77/75/75/76 (M/F=56/304) | one level | PVP(n=148)/PKP(n=97)/  SKY(n=56)/CT(n=62) | 24 months | VAS, ODI, Complications, Kyphotic angle |

**Table. S2 Scores of the Newcastle-Ottawa Quality Assessment Scale for 23 cohort studies.**

| No. | Study | Selection | Comparability | Outcome | Total |
| --- | --- | --- | --- | --- | --- |
| 1 | Lovi 2009 | ★★★★ | ★ | ★★★ | ★★★★★★★★ |
| 2 | Schofer 2009 | ★★★★ | ★★ | ★★ | ★★★★★★★★ |
| 3 | Kumar 2010 | ★★★ | ★★ | ★★★ | ★★★★★★★★ |
| 4 | Movrin 2010 | ★★★ | ★ | ★★★ | ★★★★★★★ |
| 5 | Dong 2012 | ★★ | ★★ | ★★★ | ★★★★★★★ |
| 6 | Hu 2018 | ★★★★ | ★★ | ★★ | ★★★★★★★★ |
| 7 | Zhou 2022 | ★★ | ★★ | ★★ | ★★★★★★ |
| 8 | Jindal 2022 | ★★★★ | ★★ | ★ | ★★★★★★★ |
| 9 | Diamond 2006 | ★★★★ | ★★ | ★★ | ★★★★★★★★ |
| 10 | Wang 2010 | ★★ | ★★ | ★★★ | ★★★★★★★ |
| 11 | Folman 2011 | ★★★★ | ★★ | ★★ | ★★★★★★★★ |
| 12 | Thaler 2013 | ★★ | ★ | ★★ | ★★★★★ |
| 13 | Lin 2016 | ★★★ | ★★ | ★★★ | ★★★★★★★★ |
| 14 | Huang 2020 | ★★★ | ★★ | ★★ | ★★★★★★★ |
| 15 | Lee 2012 | ★★★★ | ★ | ★★★ | ★★★★★★★★ |
| 16 | Hoshino 2018 | ★★★★ | ★★ | ★ | ★★★★★★★ |
| 17 | Yu 2022 | ★★★★ | ★★ | ★ | ★★★★★★★ |
| 18 | Pflugmacher 2012 | ★★ | ★★ | ★★ | ★★★★★★ |
| 19 | Bornemann 2017 | ★★ | ★★ | ★★★ | ★★★★★★★ |
| 20 | Otten 2013 | ★★★ | ★★ | ★ | ★★★★★★ |
| 21 | Schutzenberger 2018 | ★★★ | ★★ | ★★★ | ★★★★★★★★ |
| 22 | Bornemannet 2012 | ★★ | ★★ | ★ | ★★★★★ |
| 23 | Ee 2015 | ★★★ | ★★ | ★★★ | ★★★★★★★★ |

**Table. S3 Node splitting analyses of VAS.**

| Intervention | Direct Effect | | Indirect Effect | | Overall | | P-Value |
| --- | --- | --- | --- | --- | --- | --- | --- |
|  | Mean | Se | Mean | Se | Mean | Se |  |
| **Short-term** | | | | | | | |
| NSM, PKP | -1.3761 | 0.2308 | -1.2319 | 0.2151 | -0.1442 | 0.3152 | 0.647 |
| NSM, PVP | -1.2774 | 0.1745 | -1.5464 | 0.2552 | 0.2689 | 0.3088 | 0.384 |
| NSM, TVA | -2.3 | 0.7583 | -1.4191 | 0.2079 | -0.8808 | 0.7863 | 0.263 |
| PKP, PVP | -0.0443 | 0.1498 | -0.1053 | 0.2123 | 0.061 | 0.2599 | 0.814 |
| PKP, TVA | -0.2844 | 0.2074 | -0.0278 | 0.2555 | -0.2566 | 0.3292 | 0.436 |
| PVP, TVA | 0.0831 | 0.2291 | -0.3292 | 0.2346 | 0.4124 | 0.328 | 0.209 |
| **Long-term** | | | | | | | |
| NSM, PKP | -0.6942 | 0.2531 | -0.9307 | 0.2583 | 0.2364 | 0.3615 | 0.513 |
| NSM, PVP | -0.8608 | 0.1831 | -0.6204 | 0.3117 | -0.2403 | 0.3613 | 0.506 |
| PKP, PVP | 0.0627 | 0.1919 | -0.098 | 0.2773 | 0.1607 | 0.3371 | 0.634 |
| PKP, TVA | -0.0371 | 0.4098 | -0.1926 | 0.4716 | 0.1554 | 0.6247 | 0.803 |
| PVP, TVA | -0.1948 | 0.4423 | -0.0348 | 0.4411 | -0.1599 | 0.6248 | 0.798 |
| VAS=the Visual Analogue Score; NSM=non-surgical management; PKP=Percutaneous kyphoplasty; PVP=Percutaneous vertebroplasty; TVA=Third-generation vertebral augmentation system | | | | | | | |

**Table. S4 Node splitting analyses of ODI.**

| Intervention | Direct Effect | | Indirect Effect | | Overall | | P-Value |
| --- | --- | --- | --- | --- | --- | --- | --- |
|  | Mean | Se | Mean | Se | Mean | Se |  |
| **Short-term** | | | | | | | |
| NSM, PKP | -5.5954 | 1.8765 | -9.0389 | 1.9163 | 3.4435 | 2.6814 | 0.199 |
| NSM, PVP | -6.8297 | 1.5108 | -3.3695 | 2.218 | -3.4601 | 2.6809 | 0.197 |
| PKP, PVP | 2.0935 | 1.2335 | -0.2259 | 2.2104 | 2.3194 | 2.5319 | 0.36 |
| PKP, TVA | 2.0738 | 2.3174 | -0.8842 | 4.82 | 2.9581 | 5.3474 | 0.58 |
| PVP, TVA | -2.3 | 4.6901 | 0.6606 | 2.5689 | -2.9606 | 5.3476 | 0.58 |
| **Long-term** | | | | | | | |
| NSM, PKP | 0.16 | 5.5052 | -9.3815 | 4.152 | 9.5415 | 6.8954 | 0.166 |
| NSM, PVP | -9.1436 | 2.7645 | 0.4543 | 6.29 | -9.5979 | 6.8708 | 0.162 |
| PKP, PVP | 0.9605 | 3.2925 | -7.834 | 5.0399 | 8.7945 | 6.0195 | 0.144 |
| PKP, TVA | -8.3176 | 2.9869 | -5 | 9.2813 | -3.3175 | 9.749 | 0.734 |
| PVP, TVA | -3.7 | 8.7797 | -6.9774 | 4.2514 | 3.2774 | 9.7548 | 0.737 |
| ODI=the Oswestry disability index; NSM=non-surgical management; PKP=Percutaneous kyphoplasty; PVP=Percutaneous vertebroplasty; TVA=Third-generation vertebral augmentation system | | | | | | | |

**Table. S5 Node splitting analyses of ED-5Q.**

| Intervention | Direct Effect | | Indirect Effect | | Overall | | P-Value |
| --- | --- | --- | --- | --- | --- | --- | --- |
|  | Mean | Se | Mean | Se | Mean | Se |  |
| **Short-term** | | | | | | | |
| NSM, PKP | 0.1344 | 0.0272 | 0.031 | 0.0175 | 0.1034 | 0.0324 | 0.001 |
| NSM, PVP | 0.0358 | 0.0095 | 0.1393 | 0.031 | -0.1034 | 0.0324 | 0.001 |
| PKP, PVP | 0.0048 | 0.0147 | -0.0986 | 0.0288 | 0.1034 | 0.0324 | 0.001 |
| PKP, TVA | 0.07 | 0.0601 | -0.1425 | 20.5366 | 0.2125 | 20.5367 | 0.992 |
| **Long-term** | | | | | | | |
| NSM, PKP | 0.1003 | 0.0235 | 0.0553 | 0.0277 | 0.0449 | 0.0363 | 0.216 |
| NSM, PVP | 0.059 | 0.022 | 0.104 | 0.0288 | -0.0449 | 0.0363 | 0.217 |
| PKP, PVP | 0.0037 | 0.0167 | -0.0412 | 0.0322 | 0.0449 | 0.0363 | 0.216 |
| PKP, TVA | 0.0879 | 0.0265 | -0.168 | 5.6991 | 0.2559 | 5.6991 | 0.964 |
| ED-5Q=the EuroQol-5-Domain questionnaire; NSM=non-surgical management; PKP=Percutaneous kyphoplasty; PVP=Percutaneous vertebroplasty; TVA=Third-generation vertebral augmentation system | | | | | | | |

**Table. S6 Node splitting analyses of AVB.**

| Intervention | Direct Effect | | Indirect Effect | | Overall | | P-Value |
| --- | --- | --- | --- | --- | --- | --- | --- |
|  | Mean | Se | Mean | Se | Mean | Se |  |
| **Restoration** | | | | | | | |
| NSM, PKP | 2.3962 | 0.7738 | 6.0861 | 1.4074 | -3.6898 | 1.6045 | 0.021 |
| NSM, PVP | 12 | 2.4399 | 1.0666 | 0.6805 | 10.9333 | 2.533 | 0.0001 |
| NSM, TVA | 3 | 1.5029 | 2.6849 | 1.5746 | 0.315 | 2.1767 | 0.885 |
| PKP, PVP | -1.8121 | 0.6935 | -0.7267 | 1.4812 | -1.0854 | 1.6493 | 0.51 |
| PKP, TVA | -1.6959 | 0.568 | 0.1841 | 0.8611 | -1.88 | 1.0163 | 0.064 |
| PVP, TVA | 2.0044 | 1.2578 | -0.2747 | 0.9649 | 2.2792 | 1.586 | 0.151 |
| **Maintenance** | | | | | | | |
| NSM, PKP | 5.7 | 0.9307 | 0.8673 | 25.8403 | 4.8326 | 25.857 | 0.852 |
| PKP, PVP | -2.0374 | 0.407 | -2.0418 | 1.3906 | 0.0043 | 1.4497 | 0.998 |
| PKP, TVA | -1.5675 | 0.4183 | -1.5708 | 1.3872 | 0.0033 | 1.4496 | 0.998 |
| PVP, TVA | 0.4699 | 1.3265 | 0.47 | 0.5854 | -0.00002 | 1.4499 | 1 |
| AVB= anterior vertebral height; NSM=non-surgical management; PKP=Percutaneous kyphoplasty; PVP=Percutaneous vertebroplasty; TVA=Third-generation vertebral augmentation system | | | | | | | |

**Table. S7 Node splitting analyses of Complications.**

| Intervention | Direct Effect | | Indirect Effect | | Overall | | P-Value |
| --- | --- | --- | --- | --- | --- | --- | --- |
|  | Coef. | Std.Err | Coef. | Std.Err | Coef. | Std.Err |  |
| **AVF** | | | | | | | |
| NSM, PKP | 1.123 | 0.5429 | 0.4463 | 0.6575 | 0.6767 | 0.8529 | 0.428 |
| NSM, PVP | 0.6606 | 0.5113 | 1.3367 | 0.6854 | -0.6761 | 0.8528 | 0.428 |
| PKP, PVP | 0.3705 | 0.4803 | -0.3547 | 0.5409 | 0.7253 | 0.7243 | 0.317 |
| PKP, TVA | -0.6385 | 0.411 | -0.278 | 0.8221 | -0.3605 | 0.9189 | 0.695 |
| PVP, TVA | -0.4067 | 0.7081 | -0.7675 | 0.5829 | 0.3607 | 0.919 | 0.695 |
| **CL** | | | | | | | |
| PKP, PVP | 0.6301 | 0.2371 | 0.4454 | 0.3689 | 0.1846 | 0.4405 | 0.675 |
| PKP, TVA | -0.2094 | 0.1761 | -0.0248 | 0.4017 | -0.1845 | 0.4405 | 0.675 |
| PVP, TVA | -0.6549 | 0.3239 | -0.8395 | 0.2978 | 0.1846 | 0.4405 | 0.675 |
| AVF=adjacent vertebral fracture; CL=cement leakage; NSM=non-surgical management; PKP=Percutaneous kyphoplasty; PVP=Percutaneous vertebroplasty; TVA=Third-generation vertebral augmentation system | | | | | | | |


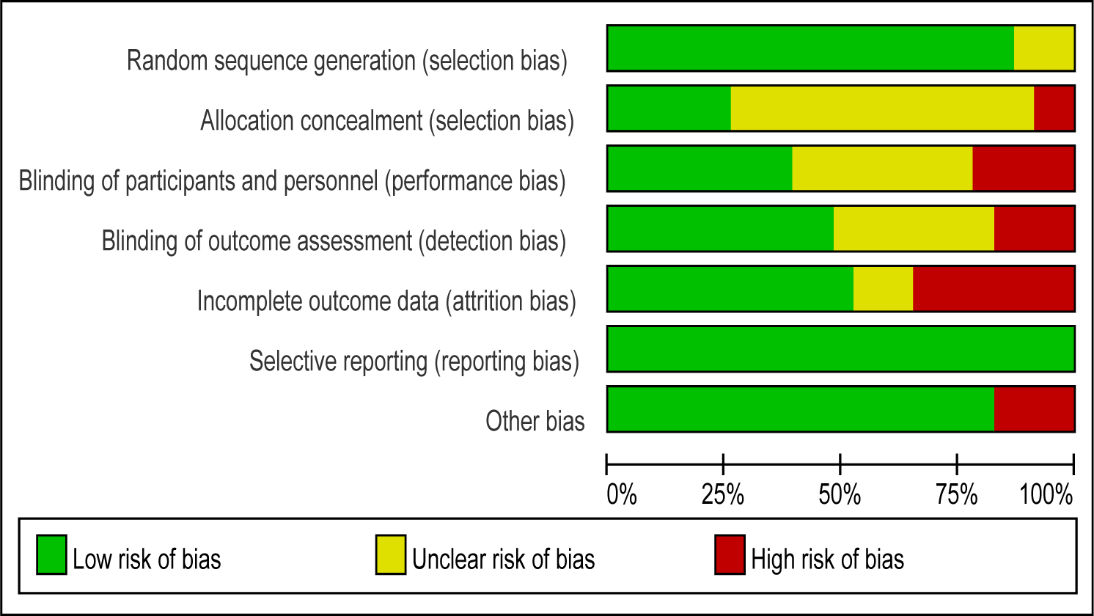


**Fig. S1 Risk of bias of included RCT studies.**


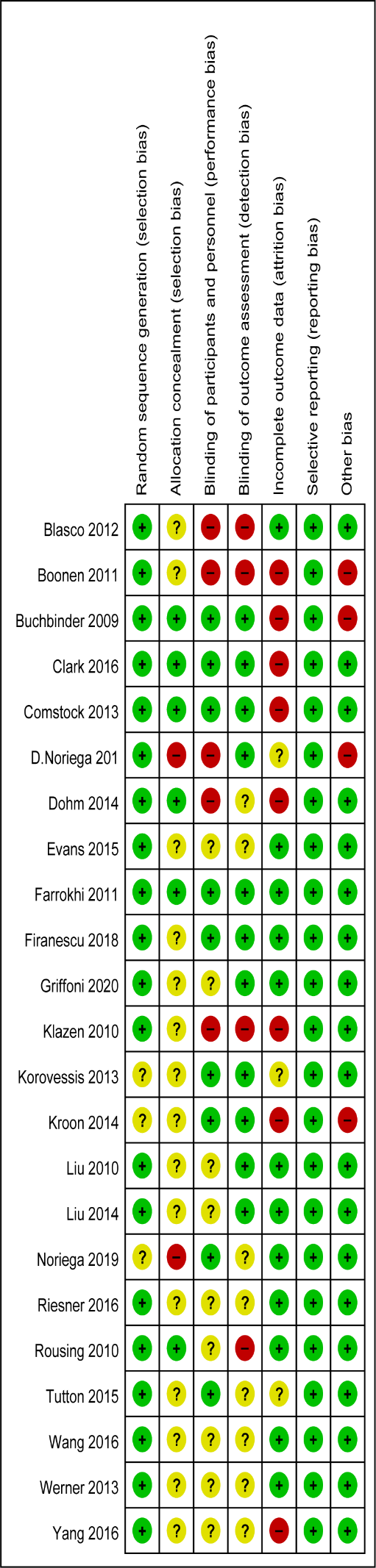


**Fig.S2 Risk of bias summary of included RCT studies.**
